# First report of immature forms of *Anopheles albitarsis s.l.* (Diptera: Culicidae) collected in artificial containers in an urban area of the state of Acre, Western Brazilian Amazon

**DOI:** 10.1590/0037-8682-0536-2025

**Published:** 2026-04-10

**Authors:** Dionatas Ulises de Oliveira Meneguetti, Gilberto Gilmar Moresco, Janis Lunier de Souza, Márcio Fernandes de Amorim, Fernanda Portela Madeira

**Affiliations:** 1 Programa de Pós-Graduação Stricto Sensu em Ciências da Saúde na Amazônia Ocidental, Universidade Federal do Acre, Rio Branco, AC, Brasil.; 2 Programa de Pós-Graduação Stricto Sensu em Ciência, Inovação e Tecnologia para Amazônia, Universidade Federal do Acre, Rio Branco, AC, Brasil.; 3 Colégio de Aplicação, Universidade Federal do Acre, Rio Branco, AC, Brasil.; 4 Secretaria de Vigilância em Saúde e Ambiente, Ministério da Saúde, Brasília, DF, Brasil.; 5 Vigilância Entomológica, Rio Branco, AC, Brasil.; 6 Universidade Federal do Acre, Centro Multidisciplinar, Campus de Cruzeiro do Sul, Cruzeiro do Sul, AC, Brasil.

**Keywords:** Mosquito-borne diseases, Malaria, Public health

## Abstract

**Background:**

This study reports the first documented occurrence of immature forms of *Anopheles albitarsis* in artificial breeding sites within an urban area of Acre, Brazil.

**Methods:**

Second-instar larvae were collected from a water tank.

**Results:**

The collected larvae were identified as *Anopheles albitarsis*, representing the first report of this species in artificial breeding sites in an urban area of Acre.

**Conclusions:**

The detection of immature forms in artificial containers suggests potential adaptation of the vector to urban settings and highlights concerns regarding the increased risk of malaria transmission in areas previously regarded as low-risk.

Malaria is a disease caused by protozoa of the genus *Plasmodium,* transmitted to humans by bites from female mosquitoes of the genus *Anopheles* ^1^. In 2023, global malaria cases were estimated at 263 million, with an incidence rate of 60.4 cases per 1,000 inhabitants, reflecting an increase of 11 million cases compared to the previous year^2^. In Brazil, malaria remains a major public health concern, particularly in the Amazon region, where more than 99% of reported cases occur^3^. 

The main malaria vectors in Brazil are members of the subgenera *Anopheles (Nyssorhynchus)* and *Anopheles (Kerteszia)*, with particular emphasis on *Anopheles (Nyssorhynchus) darlingi,* the primary vector in the Amazon region^4^ ^,^ ^5^. Approximately 103 species of Anophelinae have been documented in Brazil, including 30 yet to be formally named^3^. Within the Brazilian Legal Amazon, an estimated 49 species have been recorded^6^ ^,^ ^7^. 

*Anopheles albitarsis s.l.* (Diptera: Culicidae) represents a complex of cryptic species, comprising five formally described taxa (*Anopheles albitarsis* s.s., *Anopheles marajoara*, *Anopheles deaneorum*, *Anopheles janconnae,* and *Anopheles oryzalimnetes*), three of which (*A. marajoara*, *A. deaneorum,* and *A. oryzalimnetes*) have been documented in the state of Acre. Additionally, four other unnamed species -*Anopheles albitarsis* F, *Anopheles albitarsis* G, *Anopheles albitarsis* H, and *Anopheles albitarsis* I^8^-have been identified.

This study reports the first detection of immature forms of *A. albitarsis s.l.* collected from artificial containers in an urban area in the state of Acre, Western Brazilian Amazon. In July 2024, during routine entomological surveillance activities by the Rio Branco Municipal Health Department's entomological surveillance team, as part of the Rapid Index Survey for *Aedes aegypti* (LIRAa), larvae were collected from urban artificial breeding sites. The samples were obtained from a residence in the São Francisco housing development in Rio Branco, Acre (Lat. 9°56′52.21″S and Long. 9°56′52.21″W), located near a forest fragment and a pond, environments conducive to mosquito development. Larvae were identified in a 500-L water tank adjacent to two 1,000-L tanks (Figure 1). 

**FIGURE 1:**
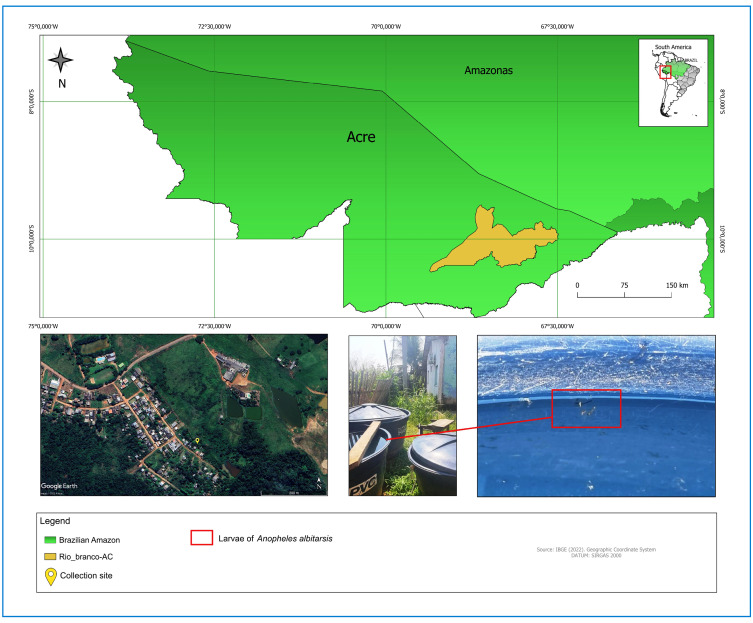
Collection site of immature *A. albitarsis s.l.* forms.

Morphological identification was carried out using the taxonomic keys of Consoli and Oliveira. The larvae were classified as second-instar *A. albitarsis s.l.* (Figure 2). Specific species determination was precluded due to limitations in infrastructure and molecular analysis resources.

**FIGURE 2:**
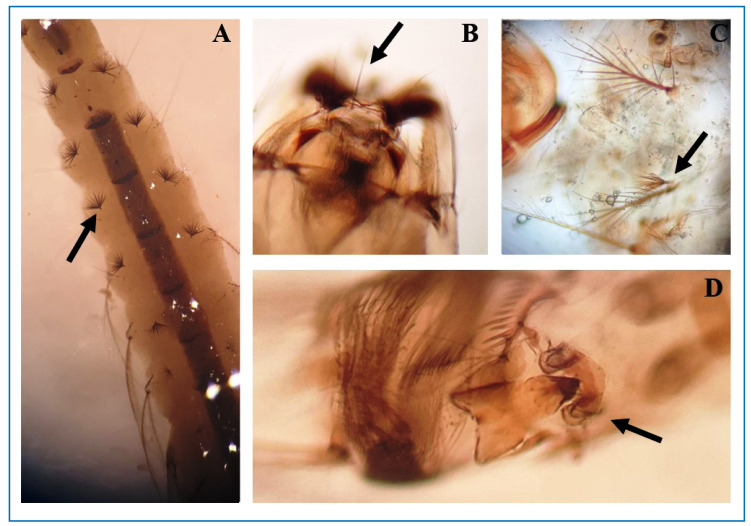
Immature forms of *A. albitarsis s.l.* collected from artificial containers in an urban area of Rio Branco, Acre. **(A)** Palmate abdominal setae with smooth branches; **(B)** Setae 2,3-C smooth and widely spaced; **(C)** Setae 2,3-P inserted on a common basal tubercle; **(D)** Spiracular plate.

Adult *A. albitarsis* exhibit abdominal tergites with postero-lateral tufts of dark scales beginning from segment III, and clear spots along the anterior wing veins, particularly on the costa, composed of pale scales similar in color to those found on posterior tarsi III-V^9^. Larval characteristics include palmate abdominal setae with smooth branches (A) and widely spaced, smooth setae 2,3-C (B). On the prothorax, setae 2,3-P arise from a common basal tubercle (C), and the presence of a spiracular plate (D)-a diagnostic feature of the genus *Anopheles* ^9^-was observed.

Research in the Brazilian Amazon has demonstrated that *A. albitarsis* can be naturally infected by *Plasmodium vivax* and *Plasmodium falciparum*, serving as an occasional vector for malaria transmission, particularly in areas where *A. darlingi* is also present^10^, such as the municipality of Rio Branco^3^. This underscores the risk of vector-borne transmission associated with this species.

The identification of immature *A. albitarsis s.l.* in artificial containers within urban settings suggests behavioral adaptation to climatic conditions, as these forms have previously been noted only in natural breeding habitats. Laporta et al.^11^ demonstrated that behavioral modifications in malaria vectors correlate with landscape changes in the Amazon. Environments with intermediate forest cover and recent human settlements are associated with increased risk of *Plasmodium* sp. transmission due to higher vector-human contact^11^.

Sampling near a forest fragment suggests possible shifts in vector behavior in response to environmental impacts of rapid, unplanned urbanization, mirroring findings from São Paulo^12^ and Rio de Janeiro^13^. Previous studies highlight that deforestation due to unregulated human expansion intensifies forest fragmentation and promotes higher vector densities, such as *Anopheles*, by increasing available breeding sites and facilitating greater human-mosquito interaction^11^ ^,^ ^14^.

Mapping breeding sites, particularly those identified in this research, aids in characterizing anopheline development in urban settings and reinforces the need to expand entomological surveillance efforts beyond traditionally recognized risk zones. Such initiatives should also inform vector control strategies, accounting for differences between *A. aegypti* and *Anopheles* spp., which pose challenges to integrated management due to their distinct behaviors^15^.

Reporting *A. albitarsis s.l.* larvae in urban artificial containers in Rio Branco constitute a novel regional finding, emphasizing the importance of robust entomological surveillance and indicating potential ecological adaptations in the species, potentially driven by environmental changes. Given its established vector competence for *Plasmodium* spp., the results highlight the need for tailored vector control programs that account for the ecological specificities of different taxonomic groups.
